# CTLA-4 and PD-1 Pathway Blockade: Combinations in the Clinic

**DOI:** 10.3389/fonc.2014.00385

**Published:** 2015-01-15

**Authors:** Margaret K. Callahan, Michael A. Postow, Jedd D. Wolchok

**Affiliations:** ^1^Melanoma and Immunotherapeutics Service, Department of Medicine, Memorial Sloan-Kettering Cancer Center, New York, NY, USA; ^2^Weill Cornell Medical College, New York, NY, USA; ^3^Ludwig Center for Cancer Immunotherapy, Memorial Sloan-Kettering Cancer Center, New York, NY, USA

**Keywords:** CLTA-4, PD-1, ipilimumab, nivolumab, immunotherapy

## Abstract

Checkpoint blocking antibodies targeting regulatory molecules on T cells such as CTLA-4 and PD-1 have reinvigorated the field of cancer immunotherapy. These agents have demonstrated clinical activity across a variety of tumor types. Now that safety and clinical activity has been demonstrated in the monotherapy setting, the field is moving in the direction of testing novel combinations.

## Introduction

Immunotherapy is poised to assume a more central role in the treatment of a variety of cancer types. The FDA-approval of the CTLA-4 blocking antibody, ipilimumab (Bristol-Myers Squibb), and the PD-1 blocking antibody, pembrolizumab (Merck) in the treatment of advanced melanoma appear to represent the proverbial tip of the iceberg. Accumulating clinical evidence points toward a promising role for checkpoint blocking antibodies in a rapidly expanding spectrum of additional solid tumors including non-small cell lung cancer, renal cell cancer, ovarian cancer, bladder cancer, head and neck cancer, and gastric cancer. While single agent CTLA-4 or PD-1 pathway blockade has demonstrated clear anti-tumor activity across multiple tumor types, responding patients are still in the minority, underscoring the importance of improving upon present options. Furthermore, in some tumors types, such as prostate cancer, single agents have a low level of activity that may be improved upon with combination approaches. Combined checkpoint blockade, to date explored with CTLA-4 and PD-1 pathway blocking agents, represents a first step in this new direction. Herein, we shall review the most up to date clinical data on these combinations, discussing both the promising clinical activity and the increased burden of toxicity seen in such combinations.

## Background

This story begins with the success of translating the basic immunologic observation that CTLA-4 is a negative regulator of T cells into the preclinical observation that blockade of CTLA-4 can have potent anti-tumor activity in mouse models, and then into the subsequent clinical trials that tested this concept in a population of patients with advanced melanoma (1–7). Two phase 3 studies have demonstrated that the human CTLA-4 blocking antibody, ipilimumab, offers a benefit in overall survival for patients with advanced melanoma, leading to the FDA-approval of ipilimumab in March 2011 (Table 1) (8, 9).

**Table 1:** **Selected clinical trials of CTLA-4 and PD-1 pathway blocking antibodies in advanced melanoma**.

Agent tested	Patients	Treatment arms	Response rates^a^	Survival
**CTLA-4 BLOCKADE**				
Ipilimumab (8)	676 patients with previously treated advanced melanoma	Ipilimumab vs. gp100 peptide vaccine vs. combination	Ipilimumab alone: ORR 10.9%	Ipilimumab alone: median OS: 10.1 months
				45.6% at 1 year
				23.5% at 2 years
		Ipilimumab dosed at 3 mg/kg every 3 weeks × 4 doses	Gp100 vaccine: ORR 1.5%	Gp100 vaccine: Median OS: 6.4 months
				25.3% at 1 year
				13.7% at 2 years
**PD-1 BLOCKADE**				
Pembrolizumab (21)	173 patients with advanced melanoma whose disease had progressed after ipilimumab	Pembrolizumab 2 mg/kg every 3 weeks vs. pembrolizumab 10 mg/kg every 3 weeks	For total study population: ORR 26%	2 mg/kg dose: 58% at 1 year
				10 mg/kg dose: 63% at 1 year
Nivolumab (20)	418 Treatment naive patients with BRAF wild-type advanced melanoma	Nivolumab 3 mg/kg every 2 weeks vs. dacarbazine	Nivolumab: ORR: 40%	Nivolumab: median OS: NR
				72.9% at 1 year
			Dacarbazine: ORR: 13.9%	Dacarbazine: median OS: 10.8 months
				42.1% at 1 year
**COMBINATION**				
Ipilimumab + nivolumab (30, 31)	52 patients with advanced melanoma (cohorts 1, 2, 2A, 3)	Multiple dose cohorts: ipilimumab 1–3 mg/kg + nivolumab 0.3–3 mg/kg	Across all dose levels: ORR: 40% (21–53%)	Across all dose levels: median OS: NR
				85% at 1 year
				79% at 2 years

*NR, not reached; OS, overall survival; ORR, objective response rate*.

*^a^ The Hodi et al. and Wolchok et al. studies used mWHO to measure response, other studies listed used RECIST criteria*.

Likewise, for PD-1, a firm foundation of basic immunologic studies, including mouse models of chronic infectious disease, helped characterize PD-1 along with its ligands PD-L1 and PD-L2, as negative regulators of effector T cell function that act predominantly in the tissue where the immune response in ongoing (10). Building upon the concept of PD-1 as a negative regulator of T cell function, subsequent studies demonstrated the potential for the PD-1 pathway to impact anti-tumor immune responses in a variety of mouse models of transplantable tumors. These studies supported the clinical development of agents that interrupt the PD-1 pathway via blockade of PD-1 itself, or one of its ligands, PD-L1. At present, numerous agents are being tested in dozens of clinical trials. At least two PD-1 blocking antibodies, pembrolizumab and nivolumab (Bristol-Myers Squibb) have demonstrated clinical activity in melanoma (Table 1), as well as several additional solid tumors including non-small cell lung cancer, renal cell cancer, ovarian cancer, and head and neck cancers (11–21). Pembrolizumab was approved by the FDA for previously treated advanced melanoma in September 2014. Three additional PD-L1 blocking antibodies have also shown clinical activity in a variety of solid tumor types: MEDI4736 (Medimmune), MPDL3280a (Genentech), and MDX-1105 (Bristol-Myers Squibb) (22–27).

Strong preclinical rationale for the clinical evaluation of combined CTLA-4 and PD-1 pathway blockade was provided by basic immunologic observations, which supported the notion that CTLA-4 and PD-1 are non-redundant pathways for the regulation of T cell responses, suggesting that the combination could have additive or synergistic potential. Furthermore, two early studies in mouse models of transplantable syngeneic tumors created further enthusiasm for this combination. The first study, presented by Korman and colleagues, demonstrated that the combination of PD-1 and CTLA-4 blockade had synergistic anti-tumor activity in a mouse model of colon adenocarcinoma, MC38 (28). In a subsequent article by Curran et al., the authors confirmed the potent anti-tumor activity of this combination when used with a cellular vaccine (Gvax or Fvax) in the B16 murine model of melanoma (29). Additionally, they found that the activity of this triple combination was associated with an increase in effector T cells in the tumor microenvironment and a relative reduction in the frequency of regulatory T cells.

## Combined PD-1 and CTLA-4 Pathway Inhibition in Advanced Melanoma

### Study design and demographics

The first study of combined checkpoint blockade tested the combination of ipilimumab with nivolumab in the treatment of advanced melanoma (Table 1) (30, 31). This small phase 1 dose-escalation study was designed to test the safety of the combination of ipilimumab at a dose of 3 mg/kg in combination with nivolumab at doses ranging from 0.3 to 3 mg/kg (cohorts 1–3). An exploratory cohort of ipilimumab dosed at 1 mg/kg in combination with nivolumab at 3 mg/kg was also included (cohort 2A). Dose limiting toxicities identified at the dose level of 3 mg/kg for both drugs identified the maximum tolerated dose for this combination. Additionally, two cohorts (6 and 7) allowed patients who had previously received commercial ipilimumab to receive nivolumab monotherapy as part of the study. Initial data on safety and response rates for this study were published in 2013 (30). Updated data on the long-term survival for the initial treatment cohorts as well as response rates and safety in an expanded number of patients treated on study (cohort 8) were reported at ASCO in 2014 (31). All of the patients treated in cohorts 1–3 and 2A were scheduled to receive concomitant doses ipilimumab and nivolumab every 3 weeks for a total of 4 doses and eligible patients who continued on treatment received the combination of both drugs every 3 months thereafter for up to 2 years. The patients treated in cohort 8 received ipilimumab at a dose of 3 mg/kg and nivolumab at a dose of 1 mg/kg. As in cohorts 1–3, patients were scheduled to receive concomitant doses of ipilimumab and nivolumab every 3 weeks for a total of 4 doses; however, subsequent dosing was on a different schedule where eligible patients who continued on treatment received nivolumab alone at a dose of 3 mg/kg every 2 weeks for up to 2 years.

### Clinical activity

The initial report of activity for the concomitant combination of ipilimumab and nivolumab was notable for objective response rate (ORR), averaging 40% (ranging from 21 to 53%, *n* = 52) across all dose-levels tested (cohorts 1–3, 2A). The inclusion of patients with stable disease for at least 24 weeks along with responders, defined as aggregate clinical activity rate, was 65% (50–83%) across all dose levels. While cross-study comparisons are inherently limited, previously reported response rates for ipilimumab monotherapy and nivolumab monotherapy were 11 and 31%, respectively (8, 11, 32). One notable feature of the responses seen for patients treated with the combination was the relatively high rate of complete responses or near complete responses; 31% of the patients treated with the concomitant combination had a reduction in disease burden of 80% or greater. Based upon this initial activity, the study was expanded and an additional 41 patients treated with the concomitant combination of ipilimumab and nivolumab (cohort 8) were reported upon at ASCO in 2014. In cohort 8, the ORR was 43 with 31% of patients showing a reduction in disease burden of 80% or greater.

As in prior studies of checkpoint blocking antibodies, the durability of responses was a notable virtue of this combination. As of reporting for ASCO 2014, responses were ongoing for the majority of responding patient and the median duration of response across all combination cohorts had not been reached. Additionally, survival data from cohorts 1–3 and 2A were presented at ASCO 2014. In aggregate, across all cohorts, the 1-year and 2-year survival was 85 and 79%, respectively. In cohort 2, the 1-year and 2-year survival was 94 and 88%, respectively.

In cohorts 6 and 7, patients who had previously been treated with ipilimumab as a monotherapy were permitted to be enrolled to receive nivolumab monotherapy at a dose of either 1 or 3 mg/kg within a 4–12 weeks window after the last dose of ipilimumab. While this was not a study requirement, the majority of patients enrolled in cohorts 6 and 7 had progressive disease after ipilimumab treatment, as assessed by their treating physician. The ORR in these two sequenced combination cohorts was 31% with all of these responses showing an 80% or greater reduction in disease burden. Clearly, some of the patients who did not have a response to ipilimumab monotherapy were able to respond to subsequent nivolumab treatment, a finding supported by a second independent study sequencing nivolumab after ipilimumab (33). One interesting observation was generated in a retrospective analysis of residual plasma levels of ipilimumab. Patients were categorized as having plasma levels of ipilimumab below or above the median plasma level of 7.255 μg/ml and the ORR seen in those with low plasma levels (14.3%) vs. high plasma levels (57.1%) appears to favor patients with higher levels of plasma ipilimumab who received nivolumab subsequently (31).

### Safety

Toxicities associated with CTLA-4 or PD-1 pathway blockade have been well described and include a constellation of tissue-specific inflammatory events that appear consistent with the known immune-activating mechanism of action for these agents. These toxicities have been referred to as immune related adverse events (irAEs) and may affect any number of organ systems including the gastrointestinal tract (colitis, diarrhea), the lung (pneumonitis), the endocrine system (hypophysitis, thyroiditis), the liver (hepatitis), the skin (rash, pruritus), and the eye (uveitis) among others. In rare cases, toxicities may be severe and potentially life threatening (colitis, pneumonitis); however, in most cases, irAEs are reversible when managed according to standard algorithms that make use of immunosuppressive medications such as steroids. In the phase 1 study of the combination of ipilimumab and nivolumab in advanced melanoma, the dose level of 3 mg/kg ipilimumab plus 3 mg/kg nivolumab was identified as exceeding the acceptable number of adverse events defined by the protocol. At this dose level, six patients were treated and three of these patients had grade 3 or 4 elevations in lipase that persisted for at least 3 weeks or longer. Also, noted in this cohort was one patient with grade 3/4 elevations in LFTs and one patient with grade 3/4 hypophysitis. All other dose-levels tested were identified as having acceptable levels of toxicity.

Two notable observations emerge from the data related to toxicity in this study. First, the observed toxicities all fall within the spectrum of irAEs already described for CTLA-4 or PD-1 pathway blocking agents; no distinctly new toxicities were described in this study. Second, the frequency of irAEs and the number of patients with multiple irAEs was notably higher than previously described for either monotherapy. Across all dose cohorts, 93% of patients who received the concomitant combination of ipilimumab and nivolumab had a treatment related toxicity of any grade and 53% had a grade 3/4 toxicity attributed to treatment. In the initial report of cohorts 1–3 and 2A, no treatment related deaths were observed, but in the expanded study including cohort 8, 1 treatment related death (multi-organ failure related to colitis) was seen among the 94 patients treated. By comparison, for ipilimumab monotherapy, irAEs of any grade are observed in ~60% of patients and grade 3/4 irAEs were observed in ~15% or patients (8). For nivolumab monotherapy, the frequency of irAEs is lower still. Consist with the high frequency of irAEs, 23% (22/94) of patients treated with the concomitant combination of ipilimumab and nivolumab discontinued treatment due to toxicities. Nevertheless, many of the patients who discontinued treatment had ongoing, durable responses that extended beyond ongoing treatment.

### Biomarkers

The search for biomarkers that might help select a patient population most likely to benefit from checkpoint blockade is an area of ongoing research, but to date, no clinically applicable biomarker appropriate for patient selection has been identified. For PD-1 and PD-L1 blocking antibodies, expression of PD-L1 in the tumor microenvironment has been intensely studies as a potential biomarker. In a small study (*n* = 9) from a single dose phase 1 study of nivolumab, Brahmer et al. reported that 3/4 patients whose tumor expressed PD-L1 on the cell surface responded to nivolumab whereas none of the remaining patients that tested negative for PD-L1 responded to the drug (34). In a subsequent analysis of a larger population of 42 patients, again, responses were restricted to those patients (9/25) that tested positive for tumor PD-L1 expression whereas none of the patients that tested negative (0/17) responded to nivolumab (11). Numerous subsequent studies have evaluated the potential for PD-L1 expression in the tumor microenvironment to predict response to PD-1 or PD-L1 blocking agents. Collectively, the data seen in studies of nivolumab, pembrolizumab, MEDI4736, and MPDL3280a confirm that PD-L1 expression is a generally favorable feature associated with a higher rate of response to PD-1 pathway blockade [reviewed in Ref. (35)]. However, another clear message also emerges from this data; some patients who test negative for PD-L1 by the assays presently in use do respond to PD-1 blocking agents, albeit at a lower rate than their PD-L1 positive counterparts. Several limitations to these studies remain to be resolved and explored including a lack of cross-study assay validation. However, given the present status of the development of this biomarker, the lack of negative predictive value for this assay precludes patient selection at this time.

PD-L1 tumor expression as a potential biomarker was explored in the present study for patients treated with the concurrent combination of ipilimumab and nivolumab (cohorts 1–3, 2A) and for those who received nivolumab monotherapy sequenced after prior ipilimumab (cohorts 6 and 7) (30). For those patients treated in the sequenced cohorts, the trend for PD-L1 expression followed prior experience for nivolumab monotherapy; an ORR of 50% (4/8) was seen in patients that tested PD-L1 positive compared to an ORR of 8% (1/13) for those that were PD-L1 negative. In notable contrast, for those patients that were treated with the concurrent combination, the ORR was relatively equivalent between those patients that were PD-L1 positive (46%, 6/13) and those that tested negative (41%, 9/22). Thus, pre-treatment PD-L1 tumor expression seems to have little predictive value in the setting of combination treatment, a finding that appears to be best explained by the relatively high rate of response to the combination seen in patients with PD-L1 negative tumors. Of course, testing for PD-L1 expression in a pre-treatment biopsy sample provides only a single static evaluation of PD-L1 expression and fails to capture dynamic changes in the PD-L1 expression that may accompany these treatments. Additional studies looking at pharmacodynamic changes in the peripheral blood after treatment with ipilimumab and nivolumab demonstrated robust changes in immune activation markers such as ki67 and ICOS as well as upregulation of checkpoint molecules PD-1 and CTLA-4 (36). In this small sample size, these pharmacodynamics changes did not correlate with clinical outcomes, but additional studies are ongoing exploring changes in peripheral blood and tumor markers in this setting.

Appropriate caution should be applied to the results of a small, non-randomized phase 1 study conducted at a small number of academic sites. The true measure of the relative benefit, and toxicity, of this combination approach will be best established in larger, randomized studies. These studies are better positioned to address the outstanding questions of the relative benefits of concomitant vs. sequential therapies with these agents and will better assess the relative merits and liabilities of a combination regimen with an apparently higher toxicity burden. A randomized two-arm phase 2 study comparing the combination to ipilimumab monotherapy (NCT01927419) and a randomized three-arm phase 3 study comparing the combination to either nivolumab monotherapy or ipilimumab monotherapy (NCT01844505) have completed accrual and results are awaited.

## Combined PD-1 and CTLA-4 Pathway Inhibition in RCC (37)

### Study design and demographics

The data reported by Dr. Hammers at the ASCO meeting in June of 2014 reflect two selected cohorts of a larger study, CA209-016 (NCT01472081) where nivolumab was tested in combination with TKI’s or ipilimumab for the treatment of metastatic RCC. Dr. Hammers presented data on the two cohorts involving the combination of ipilimumab and nivolumab, either at the dose levels of 3 mg/kg nivolumab plus 1 mg/kg ipilimumab (N3 + I1) or at the dose levels of 1 mg/kg nivolumab plus 3mg/kg ipilimumab (N1 + I3), with 21 and 23 patients, respectively, in each of these cohorts. The primary endpoint of the study was safety and the secondary endpoints were ORR, duration of response, and progression free survival. In contrast to the previously report study in melanoma, tumor response was assessed by RECIST v 1.1 criteria and the first tumor assessment was performed at 6 weeks. The treatment schema was comparable to the schedule utilized in cohort 8 of the melanoma study; the combination was given every 3 weeks for up to for doses (so called induction) followed by nivolumab monotherapy administered every 2 weeks, or a maintenance phase.

For a relatively small study, the two arms, N3 + I1 and N1 + I3 were relatively balanced, although some differences in prior treatment regimens were noted. For example, 57% of the patients in the N3 + I1 arm received prior IL-2 therapy, whereas only 26% of patient in the N1 + I3 arm did. Conversely, prior treatment with antiangiogenic therapies (65 vs. 48%) and mTOR inhibitor therapies (30 vs. 24%) was more common in the N1 + I3 arm. In both arms, ~20% of the patients were treatment naïve when they entered the study. It is unclear what impact, if any, prior therapies may have had on the clinical activity or toxicity seen in the nivolumab plus ipilimumab combination.

### Safety

Both combination arms were found to be safe, and no grade 5 treatment related AEs were reported. Nevertheless, treatment related toxicities were common in both arms, with 76% (N3 + I1) and 100% (N1 + I3) of patients experiencing a toxicity of any grade. There was an apparent difference in the frequency of grade 3–4 treatment AEs between the two cohorts with only 29% (*n* = 6) of patients in the N3 + I1 cohort experiencing these events compared to 61% (*n* = 14) of patients in the N1 + I3 cohort. Similar to the melanoma experience, asymptomatic laboratory abnormalities such as elevations in AST, ALT, amylase, and lipase comprised the largest portion of grade 3–4 events. Grade 3–4 diarrhea was observed in both the N3 + I1 arm (5%) and the N1 + I3 arm (13%). No cases of grade 3–4 pneumonitis were observed in this study at the time of reporting.

### Clinical activity

Significant clinical activity for the combination of nivolumab and ipilimumab was observed in both cohorts with confirmed ORRs of 43% (N3 + I1) and 48% (N1 + I3). Most of the responses were ongoing at the time data were reported, 78% in the N3 + I1 arm and 82% in the N1 + I3 arm. Likewise progression free survival was similar between N3 + I1 and N1 + I3 arms, with 24 weeks PFS at 65 and 64%, respectively. With the important caveat that cross-study comparisons should be taken as hypothesis generating, the response rates seen with the combination of ipilimumab and nivolumab compare favorably to those reported for a phase 2 study of nivolumab as a monotherapy, where response rates of 20, 22, and 20% were seen across dose cohorts of 0.3, 2, and 10 mg/kg, respectively. An important limitation in this comparison is that all patients in the phase 2 study were previously treated with 1–3 prior therapies including at least 1 antiangiogenic agent.

### Biomarkers

The biomarker, tumor PD-L1 was also tested in this small study of RCC. Very consistent with the experience in melanoma, in patients treated with the combination of ipilimumab and nivolumab, PD-L1 status appears unrelated to response rate. This stands in contrast to the experience with PD-L1 status in patients treated with PD-1 blockade alone.

## Combined PD-1 and CTLA-4 Pathway Inhibition in NSCLC (38)

### Study design and demographics

The abstract reported by Dr. Antonio at the ASCO meeting in June of 2014 reflected two selected cohorts of a larger study, CA209-012 (NCT01454102) where nivolumab combinations were tested for the treatment of squamous or non-squamous advanced NSCLC. Dr. Antonio presented data on the two cohorts involving the combination of ipilimumab and nivolumab, either at the dose levels of 3 mg/kg nivolumab plus 1 mg/kg ipilimumab (N3 + I1) or at the dose levels of 1 mg/kg nivolumab plus 3 mg/kg ipilimumab (N1 + I3), with 25 (9 squamous, 16 non-squamous) and 24 (9 squamous, 14 non-squamous) patients, respectively, in each these cohorts. The primary endpoint of the study was safety and tolerability and the secondary endpoints were ORR and progression free survival at 24 weeks. As in the RCC study, tumor response was assessed by RECIST v 1.1 criteria; the first tumor assessment was performed at 10 weeks. The treatment schema was comparable to the schedule utilized in cohort 8 of the melanoma study; the combination was given every 3 weeks for up to for doses (so called induction) followed by nivolumab monotherapy administered every 2 weeks, or a maintenance phase.

### Safety

Treatment related toxicities were common across all arms of the study with an 88% aggregate rate of toxicities of any grade, 78% in the squamous N1 + I3 arm, 100% in the non-squamous N1 + I3 arm, 78% in the squamous N3 + I1 arm, and 88% in the non-squamous N3 + I1 arm. A number of serious, grade 3–4 adverse events were reported including pneumonitis (6%), diarrhea/colitis (16%), rash (4%), endocrinopathies (6%) nephritis (2%), and hepatitis (8%). Three deaths that were due to drug-related toxicities were reported including 1 case each of respiratory failure following grade 3 colitis, pulmonary hemorrhage, and toxic epidermal necrolysis in a patient with a history of colitis.

### Clinical activity

Clinical activity for the combination of nivolumab and ipilimumab was relatively modest across cohorts in this small study of NSCLC, with an aggregate ORR of 16%. Response rates in each individual arm were 11% (squamous, N1 + I3), 13% (non-squamous N1 + I3), 33% (squamous, N3 + I1), and 13% (non-squamous, N3 + I1). PFS at 24 weeks was 41% for patients in the N1 + I3 arm and 29% for patients in the N3 + I1 arm. As reported previous in both melanoma and RCC, PD-L1 status appeared to have no clear predictive value in patients with NSCLC treated with the combination of ipilimumab and nivolumab.

## Outstanding questions

The combination of ipilimumab and nivolumab is just the first step in exploring the utility of combined checkpoint blockade. Further studies testing this combination in a variety of tumor types are ongoing. Additional approaches to testing combined CTLA-4 and PD-1 pathway blockade, including the combination of tremelimumab and MEDI4736 are also underway. As new agents become available, including checkpoint blocking antibodies against LAG-3, Tim-3, among other targets, new opportunities for combinations will abound. Despite preclinical data in mouse models, it is unclear which combinations, in which disease types and at which doses/schedules will have the greatest impact on human cancers. We await additional clinical data to guide these choices.

## Conflict of Interest Statement

Margaret K. Callahan and Jedd D. Wolchok both receive research support from Bristol Myers Squibb. Michael A. Postow as no conflicts of interest to declare.
